# Multi-Contrast Differentiation by Dual-Energy Spectral CT Angiography in a Patient with Pulmonary Barium Granulomas

**DOI:** 10.3390/diagnostics13050832

**Published:** 2023-02-22

**Authors:** Tommaso D’Angelo, Francesco M. Arico, Lydia Broccio, Giorgio Ascenti, Silvio Mazziotti, Christian Booz, Simon S. Martin, Ibrahim Yel, Ludovica R. M. Lanzafame, Alfredo Blandino, Carmelo Sofia

**Affiliations:** 1 Diagnostic and Interventional Radiology Unit, BIOMORF Department, University Hospital “Policlinico G. Martino”, 98124 Messina, Italy; 2 Department of Radiology and Nuclear Medicine, Erasmus MC, 3015 GD Rotterdam, The Netherlands; 3 Division of Experimental Imaging, Department of Diagnostic and Interventional Radiology, University Hospital Frankfurt, Theodor-Stern-Kai 7, 60590 Frankfurt am Main, Germany

**Keywords:** computed tomography angiography, respiratory tract fistula, pneumonia, aspiration, contrast media, barium

## Abstract

Barium inhalation usually relates to accidental aspiration during radiological procedures with an oral contrast agent. When present, barium lung deposits are visible as high-density opacities on chest X-ray or CT scan due to high atomic number, and they may be indistinguishable from calcifications. Dual-layer spectral CT has shown good material differentiation capabilities, due to its increased high-Z element range and smaller spectral separation between low- and high-energy spectral data. We present the case of a 17-year-old female with a history of tracheoesophageal fistula, who underwent chest CT angiography on a dual-layer spectral platform. Despite the close Z numbers and K-edge energy levels of the two different contrast materials, spectral CT was able to identify barium lung deposits from a previous swallowing study and to clearly distinguish them from calcium and the surrounding iodine-containing structures.

## 1. Introduction

The term “*baritosis*” refers to benign pneumoconiosis caused by the inhalation of barium. Firstly reported by Fiori in 1926, it has been described in the last century in association with chronic exposure to barium dust resulting from paint manufacturing [1]. Nowadays, the presence of barium deposits within the lungs is usually related to accidental aspiration during radiological procedures with an oral barium-containing contrast agent. Despite being infrequent, this event is well known and its occurrence increases when predisposing factors are present, such as all the causes leading to dysphagia (e.g., head and neck cancers, cerebrovascular accidents, closed head or spinal cord injuries, progressive neurologic disease, Zenker’s diverticulum, esophageal strictures) [2]. The presence of a tracheoesophageal fistula (TEF) also represents an intuitive cause of barium aspiration, despite this being poorly documented in the scientific literature [3,4]. Aspirated barium may accumulate within the lungs resulting in multiple millimetric nodules visible on chest X-ray or CT scan due to the high atomic number (Z = 56). These high-density opacities can persist for multiple years since the clearance from the alveolar air space is very slow and may be indistinguishable from lung calcifications [5]. Spectral CT is an increasingly consolidated imaging technique that allows one to obtain high- and low-energy data from CT scans, providing qualitative and quantitative information about the tissue composition [6,7,8,9,10,11,12]. To the best of our knowledge, this is the first case to report a comprehensive CT assessment of barium granulomas based on spectral material decomposition algorithms.

## 2. Case Report

**Figure 1:**
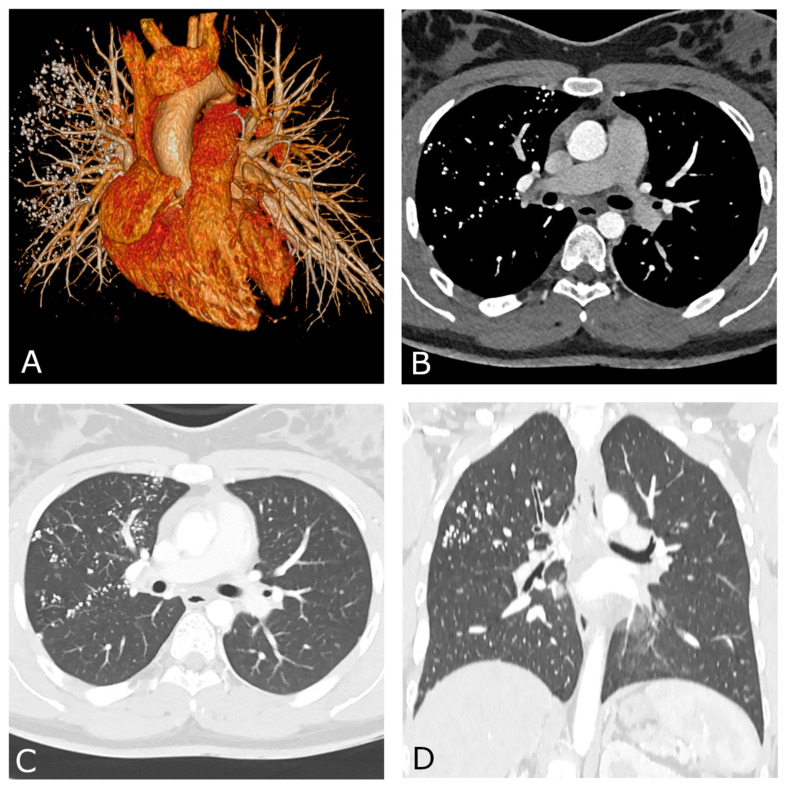
Chest CT angiography with ECG gating performed on a dual-layer DECT platform. Volume-rendering reconstruction (**A**) shows normal cardiovascular anatomy with multiple high-density foci within the right lung. Both axial (**B**,**C**) and coronal reconstructions (**D**) demonstrate the typical perilymphatic distribution of the lung nodules. Mediastinal (**B**) and lung parenchyma (**C**) views performed along the same axial plane are shown.

A 17-year-old female, with a history of surgical treatment for congenital Type C esophageal atresia at birth, and a diagnosis of distal tracheoesophageal fistula (TEF) relapse, came to us for observation for pre-operative planning. The patient had mild dysphagia to solid foods but was otherwise healthy. She did not have respiratory symptoms or coughing during meals. Chest CT angiography (CTA) with ECG triggering was performed to exclude any congenital cardiovascular abnormalities prior to surgery. The patient received 1.2 mL/kg body weight of intravenous nonionic contrast medium (Iomeron 400, Bracco, Milan, Italy) followed by 40 cc of diluted contrast bolus, using a power injector system at a rate of 5 mL/s through an 18-gauge i.v. catheter placed in the right arm. The scan was performed on a dual-layer dual-energy CT (DECT) platform (IQon Spectral CT, Philips Healthcare, Best, The Netherlands) only during the arterial phase, to minimize the radiation exposure. Chest CTA did not reveal any cardiovascular abnormalities. As a collateral finding, multiple high-density foci were noted within the right lung (Figure 1). They were located in almost all segments of the right upper lobe and in the middle lobe with a mainly perylimphatic distribution. These lesions were partly confluent and ranged in size from 1 to 6 mm. Moreover, confined “tree-in-bud” opacities and thickened interlobar septa were also noted. No signs of bronchial distortions, honeycombing or other fibrotic-like changes were present.

**Figure 2:**
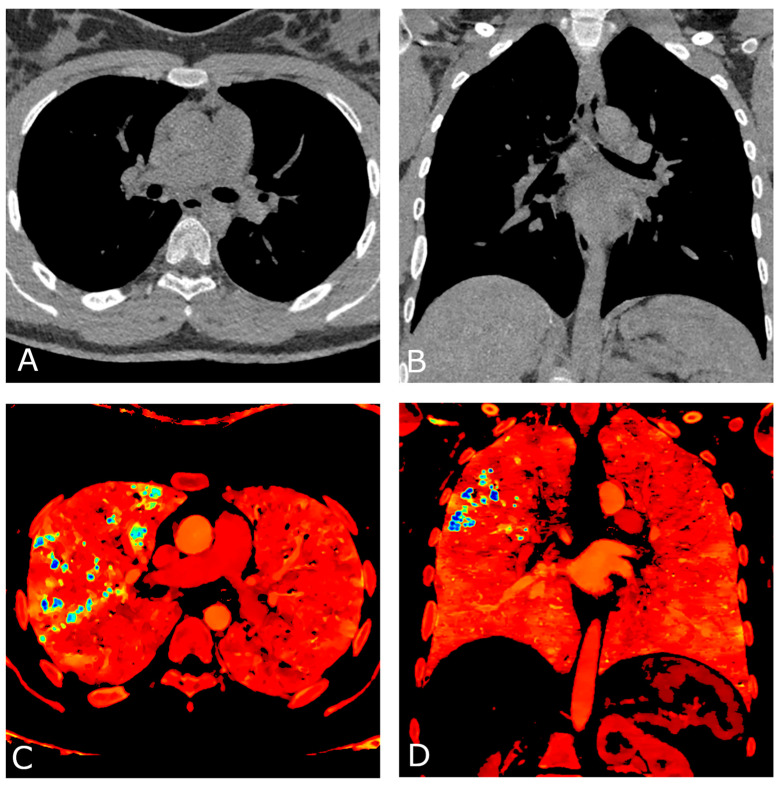
Chest CT angiography with ECG gating performed on a dual-layer DECT platform. Axial (**A**) and coronal (**B**) virtual non-contrast reconstruction (VNC) images. All pulmonary lesions are removed along with the iodinated contrast medium, suggesting similar Z number of their composition material with the iodinated contrast agent. Axial (**C**) and coronal (**D**) color-coded reconstruction maps based on Z-effective number, depict higher values of the pulmonary nodules (blue) compared to iodinated structures (green), consistent with a higher Z number of barium (Z = 56) than iodine (Z = 53).

**Figure 3:**
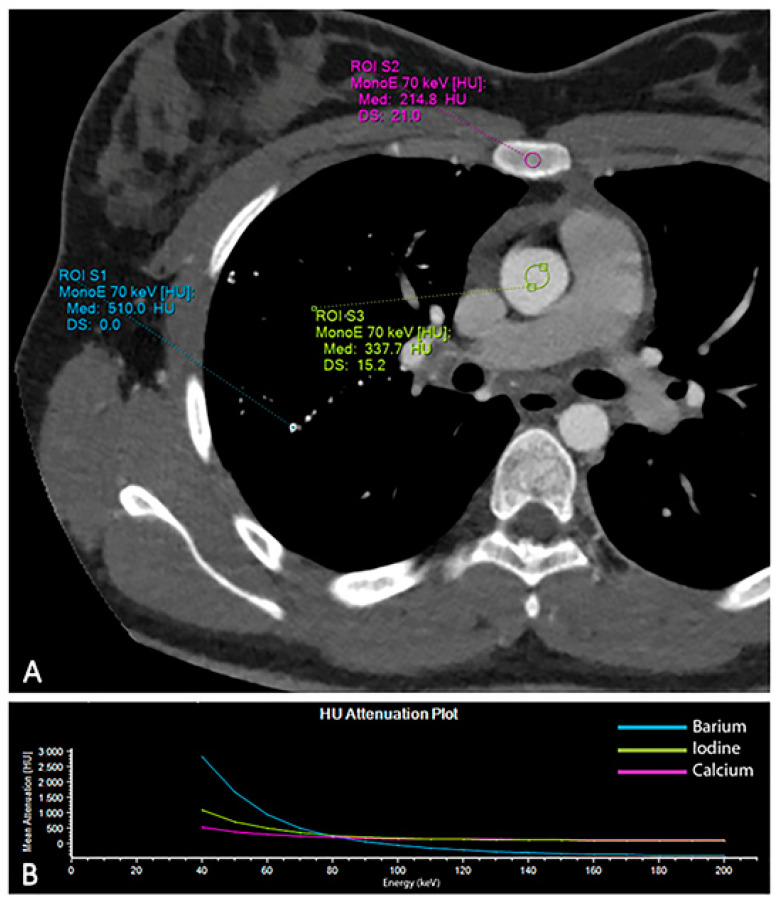
Analysis of the attenuation curves of different materials based on spectral CT data. Regions of interest were placed within the aorta (iodine), in the pulmonary micronodules (barium) and in the sternum (calcium) to investigate the attenuation curves of the different materials (**A**). The graph displays how each material has exclusive changes in attenuation at the different energy levels (**B**).

**Figure 4:**
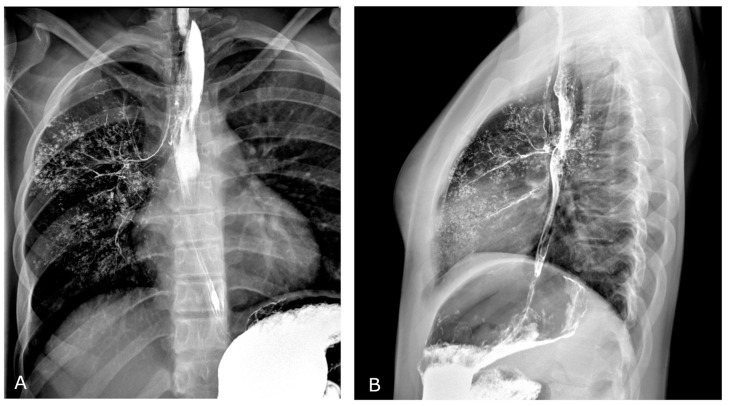
Barium-swallowing study performed two years before (study recovered from patient’s medical record and performed in a different institution). Frontal (**A**) and lateral (**B**) views show opacification of right bronchial tree due to transit of barium-containing contrast agent through a small tracheoesophageal fistula.

A comprehensive spectral evaluation was performed to characterize the high-density nodules. Firstly, virtual non-contrast reconstruction was obtained to derive the pre-contrast images (Figure 2A,B). All the lesions were removed along with the iodinated contrast medium, suggesting high Z number of the material and excluding the presence of calcium-containing lesions (Z = 20). Regions of interest were placed within the micronodules, in the sternum and in the aorta to investigate differences in attenuation (Figure 3). Despite the curves showing similar patterns, increasing at low energy levels (i.e., 40 keV), the lung nodules had the highest profile, even when compared to the iodine attenuation curve. Color-coded reconstruction maps based on Z-effective number also revealed that the lung nodules were composed of a material with higher Z number than iodine (Figure 2C,D). A careful review of the patient’s medical record showed that the patient had undergone a barium-swallowing study in another institution two years earlier. This study was interrupted due to the transit of a considerable amount of contrast material into the right bronchial tree through a small tracheoesophageal fistula (Figure 4). Patient’s medical history, together with spectral information, allowed for diagnosis of barium lung granulomas related to a previous swallowing study.

## 3. Discussion

Barium deposits resulting from aspiration usually appear as multiple millimetric high-density opacities on chest X-ray or CT scan due to the high atomic number (Z = 56) [5]. The pulmonary regions involved depend on the position of the patient during and after the aspiration or on the location of TEF if present [13]. Although severe inflammatory responses and two cases of death have been reported in elderly patients [13,14], inhaled barium has proven to be a relatively inert material; the initial symptoms and effects on respiratory function are usually mild and severe lung damage is not usually expected [1,3]. After aspiration, clearance of inorganic particles from the alveolar air space may occur though different mechanisms, including phagocytosis by alveolar macrophages, deposition on alveolar or peribronchiolar interstitial tissue and transport via lymphatics in the interlobular septa and after in the pleura. Barium particles can persist within the lungs for multiple years due to the slowness of this process and the difficulty of achieving a complete clearance [5]. Akata et al. reported a case of a barium aspiration presenting with a crazy-paving appearance in the sub-acute setting [15]. In other cases, only subpleural cysts were visible, and they were considered suspicious for the presence of fibrosis precursors [5]. In our case, only subtle pulmonary findings were found two years after the barium inhalation. In particular, tree-in-bud opacities, compatible with bronchiolitic phenomena, were present in the upper lobes. On the other hand, there were no signs of bronchial distortions, honeycombing or other fibrotic-like changes. Differential diagnosis for multiple parenchymal high-density foci identified within the lungs should include pulmonary alveolar microlithiasis (PAM), a rare lung disease characterized by intra-alveolar spreads of calcification of both lungs, as well as calcium deposition due to hypercalcemia, which can result from several causes (i.e., sarcoidosis) [5,15].

Conventional CT alone is not able to reliably differentiate high-density material based solely on Hounsfield Units [16]. Conversely, DECT imaging allows one to differentiate materials via spectral analysis of tissues at different energy levels, thus providing additional information about tissue composition [17,18,19,20,21,22]. In our case, three materials with different atomic numbers (Calcium (Z = 20), Iodine (Z = 53), Barium (Z = 56)) were differentiated within a single CT scan, resulting in an improvement in diagnostic confidence and a reduction in radiation exposure for the young patient. In particular, spectral CT allowed for the differentiation of Calcium (Z = 20) from Iodine and Barium, thanks to the large discrepancy in atomic Z numbers [16,23,24]. However, it performed well even for differentiating two materials with much closer Z numbers, such as Iodine (Z = 53) and Barium (Z = 56), being able to reveal the higher values of Barium components relatively to the surrounding Iodine-containing structures in Z-effective color-coded maps (Figure 2). Our case confirms the results of Anderson et al., who were able to differentiate the two contrast materials in mice by means of spectral imaging [16]. To the best of our knowledge, this is the first case to report a comprehensive DECT assessment and differentiation between Iodine and Barium components in humans. This might be related to the recent introduction of a dual-layer DECT platform that reaches higher material differentiation capabilities in comparison to dual-source and fast kVp switching platforms, likely due to the increased high-Z element range and smaller spectral separation between low- and high-energy spectral datasets [23]. Moreover, while for most of the currently available DECT platforms, the preventive selection of dual-energy protocol is needed prior to scanning, dual-layer DECT scanners always provide spectral data without affecting the clinical workflow. This feature might be particularly useful, since spectral information can be reviewed retrospectively, especially when the diagnosis may not be straightforward. Currently, Iodine and Barium are the only contrast agents approved for X-ray and CT imaging. Our findings might represent a driver to further explore the potential of Z-effective maps for multi-contrast material differentiation in spectral CT imaging, and to speculate about their potential applications in clinical routine.

## Data Availability

Data is contained within the article.
